# Endoplasmic reticulum stress within the primary motor cortex in hypobaric hypoxia-induced acute urinary retention

**DOI:** 10.1016/j.ibneur.2025.11.009

**Published:** 2025-11-08

**Authors:** Quanchao Zhang, Yingying Ma, Caibao Lu, Ling Nie, Hongwei Chen, Jiujian Cao, Jinghong Zhao, Yinghui Huang

**Affiliations:** a Department of Nephrology, the Key Laboratory for the Prevention and Treatment of Chronic Kidney Disease of Chongqing, Chongqing Clinical Research Center of Kidney and Urology Diseases, Xinqiao Hospital, Army Medical University (Third Military Medical University), Chongqing, China; b Medical Center of Hematology, Xinqiao Hospital, State Key Laboratory of Trauma, Burn and Combined Injury, Army Medical University, Shapingba District, Chongqing, China; c 953th Hospital, Army Medical University (Third Military Medical University), Shigatse, China

**Keywords:** Acute urinary retention, Hypobaric hypoxia, Endoplasmic reticulum stress, Primary motor cortex, 4-Phenylbutyrate

## Abstract

**Purpose:**

Acute urinary retention (AUR) is a prevalent clinical challenge following rapid exposure to hypobaric hypoxia (HH). Neurons are highly sensitive to HH, and the destruction or damage of primary motor cortex (M1) neurons will lead to micturition dysfunction and subsequent urinary retention. The aim of this study is to elucidate the potential mechanism of HH induced AUR in M1.

**Methods:**

Mice were subjected to a simulated HH environment to establish AUR model. ELISA is used to detect inflammatory markers, Nissl and TUNEL staining is used to detect neuronal damage and apoptosis, and western blot is used to detect the expression of endoplasmic reticulum stress (ERS) and apoptosis related proteins.

**Results:**

Compared to the normoxic group, mice in the HH group exhibited AUR, characterized by diminished urine output and frequency and increased single voiding volume. Simultaneously, the levels of inflammatory cytokines (IL-1β, IL-6, TNF-α) also significantly increased. Nissl staining and TUNEL staining showed more severe neuronal damage and apoptosis caused by HH. Western blot results confirmed that the increased expression of pro-apoptotic markers caspase-3 and Bax, while decreased expression of anti-apoptotic marker Bcl-2, indicating an increase in neuronal apoptosis. However, administration of endoplasmic reticulum stress inhibitor 4-PBA significantly improved AUR, reduced neuroinflammation, neuronal damage and apoptosis.

**Conclusion:**

These findings confirm that ERS plays a key role in HH induced AUR.

## Introduction

Acute urinary retention (AUR) is a common clinical condition following rapid ascent to high altitudes, characterized by reduced urinary frequency, decreased urine volume, and diminished bladder contractility (Verratti et al., 2016, Wang, 2012). If left untreated, AUR can lead to irreversible bladder damage and may progress to chronic urinary retention. Although AUR associated with high-altitude exposure has been documented, its epidemiological profile remains poorly described, and the underlying mechanisms are still unclear. It is well established that hypobaric hypoxia (HH) represents a major pathogenic factor in high-altitude illnesses (Li et al., 2018). Thus, investigating the potential causal relationship between HH and AUR is of considerable interest.

Conventional understanding attributes the neural control of micturition largely to subcortical reflex circuits, which involve coordination among the lower urinary tract, spinal cord, pontine micturition center, and periaqueductal gray. These circuits detect bladder distension and initiate voiding once a critical volume is reached (Griffiths, 2015, Fowler et al., 2008a, Fowler et al., 2008b). In previous work, we identified neurons within the primary motor cortex (M1) upstream of the bladder and demonstrated for the first time a causal link between their activity and urination: activation of these neurons induces bladder contraction and initiates voiding, whereas their inhibition or ablation impairs urination and leads to urinary retention (Yao et al., 2018). However, it remains unknown whether HH induces damage in M1 neurons, thereby contributing to AUR.

The mechanisms underlying HH-induced neuronal injury are multifaceted. A key mechanism involves excitotoxicity mediated by overactivation of glutamate receptors, especially N-methyl-D-aspartate (NMDA) receptors. Chronic HH exposure has been shown to upregulate NMDA receptor expression, leading to neuronal apoptosis (Ji et al., 2021). Additionally, HH compromises mitochondrial integrity, which is critical for neuronal survival. The enzyme 12/15-lipoxygenase has been implicated in HH-induced mitochondrial dysfunction, promoting cytochrome-c release and activation of the intrinsic apoptotic pathway (Choudhary et al., 2022). Therefore, understanding the mechanisms underlying HH-induced neuronal damage in M1 is crucial for elucidating the pathogenesis of AUR.

Endoplasmic reticulum stress (ERS) represents a cellular response to external stressors that helps restore homeostasis and has been increasingly recognized in the pathogenesis of various diseases (Almanza et al., 2019). Notably, HH, as a potent environmental stressor, is known to disrupt intracellular homeostasis, triggering ERS and apoptosis (Jain et al., 2016a, Jain et al., 2016b, Cui et al., 2018a, Cui et al., 2018b). However, it is not yet clear whether HH induces ERS in neurons and thereby contributes to neuronal damage. Therefore, this study aimed to investigate whether HH impairs M1 neuronal function via ERS, leading to AUR.

## Materials and methods

### Animals

C57BL/6 J mice (2–5 months old, male) were purchased from the Laboratory Animal Center at the Army Military Medical University. Mice were group-housed (5 maximum per cage) under a 12-h light/dark cycle and provided with water and food ad libitum. All experimental procedures were approved by the Army Military Medical University Animal Care and Use Committee and performed in accordance with the Institutional Animal Welfare Guidelines. For all experiments, mice were randomly assigned to experimental and control groups.

### Experimental grouping

**Fig. 1:**
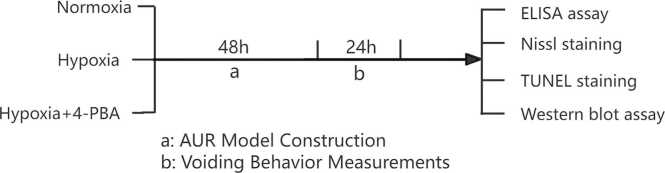
The schematic flowchart of experimental workflow.

The mice were randomly divided into three groups: the normoxia group, the hypoxia group, the hypoxia plus 4-PBA treatment group (intraperitoneal injection of 80 mg/kg). All groups received intraperitoneal injection, but with an equal volume of 0.9 % physiological saline. The specific experimental workflow are shown in Fig. 1.

### Hypobaric hypoxia acute urinary retention model

The mice in the hypoxia and the hypoxia plus 4-PBA treatment group were placed in a hypobaric chamber with an oxygen content of only 10 % (10 % represents Fraction of inspiration O_2_ (FiO_2_)) and air pressure of 364 mmhg (approximately 0.47 atmospheric pressure, or equal to 5800 m altitude above sea level) for 48 h. Differently, the mice in the hypoxia plus 4-PBA treatment group were first intraperitoneally injected with 4-PBA at a dose of 80 mg/kg before placed into the hypobaric chamber. The normoxia group mice were placed in a normoxia conditions. In all groups, urine was collected for 24 h using metabolic cages.

### Voiding behavior measurements using metabolic cages

To evaluate voiding behavior, mice were individually placed in a metabolic cage (Tecniplast Japan Co. Ltd., Tokyo, Japan) mounted on an electrical balance which was connected to a computer used for data collected. Each mouse was provided with free access to food and water. After an acclimation period of 3 days in the cage, data on voided urine were continuously collected for each mouse over 24 h.

### Tissue preparation

After the urine experiment, the mice were immediately decapitated and brain tissue was collected.

For tissue treatment for ELISA and Western blot analysis, the mice were perfused with 0.9 % NS and the micro-dissected M1 brain area was immediately detected for inflammatory factors and related proteins. For Nissl and TUNEL staining, the mice were perfused with 0.9 % NS and then with 4 % paraformaldehyde, the brains were then paraffin-embedded and sliced.

### ELISA assay

Levels of TNF-α、IL-6 and IL-1β in the M1 were measured using ELISA kits. The experiment was performed strictly under the ELISA instructions, and the concentration was normalized according to the standard curve.

### Nissl staining

Tthe sections were placed in 1 % toluidine blue aqueous solution preheated to 50°C and dyed in a 56°C temperature box for 20 min. Next, the sections were washed with distilled water, soaked in 70 % alcohol for 1 min, and treated with 95 % alcohol differentiation. Anhydrous alcohol was used for rapid dehydration followed by treatment with transparent xylene and neutral gum seal.

### TUNEL staining

Terminal dexynucleotidyl transferase(TdT)-mediated dUTP nick end labeling(TUNEL)staining was performing to detect neuronal apoptosis. Briefly, the brain sections were fixed with a 4 % paraformaldehyde solution for 0.5 h at room temperature. Next, the sections were incubated with a methanol solution containing 0.2 % H_2_O_2_ for 0.5 h to block endogenous peroxidase activity. They were then treated with the TUNEL reaction mixture (Roche, Germany), then the brain sections were maintained in a 37 °C incubator for 60 min. The results are presented as the apoptosis indices, which was quantified as (TUNEL-positive cells)/(total cells)× 100 %.

### Western blot assay

The peri-infarct brain tissues were homogenized and lysed with RIPA buffer (Thermo Scientific™, Chelmsford, MA, USA) supplemented with protease and phosphatase inhibitor cocktails (Abcam, Cambridge,MA, USA). Proteins (20 μg) were separated by SDS-PAGE and transferred to PVDF membranes (Millipore, Shanghai, China). After blocking with 5 % nonfat milk, the PVDF membranes were incubated with primary antibodies in the cold room overnight. After being washed with the Tris-buffered saline-Tween 20 (TBST) buffer three times, the PVDF membranes were incubated with peroxidase-conjugated secondary antibodies. The membranes were then washed with TBST three times, and the proteins were visualized with an ECL detection solution (Millipore, Shanghai, China). The primary antibodies used were as follows: GRP78 (Mouse #66574–1-Ig, 1:3000, Proteintech), CHOP (Mouse #ab11419, 1:1000, Abcam), GAPDH (Mouse #60004–1-Ig, 1:3000, Proteintech), anti-Bax (Cat #2774,1:1000, Cell Signaling Technology, Beverly, MA, USA), anti-Bcl-2 (Cat #3498, 1:1000, Cell Signaling Technology, Beverly, MA, USA), anti-caspase-3 (Cat #4150, 1:1000, Cell Signaling Technology). The intensity of the protein bands was quantified with ImageJ software and normalized to that of GAPDH. Each test was repeated at least three times.

### Statistical analysis

Statistical analyses were performed using SPSS software (version 23.0). All results were analyzed using one-way ANOVA, followed by Fisher's test to assess differences between groups. P value of< 0.05 is considered significant.

## Results

### Hypobaric hypoxia induces urinary retention and neuroinflammation

**Fig. 2:**
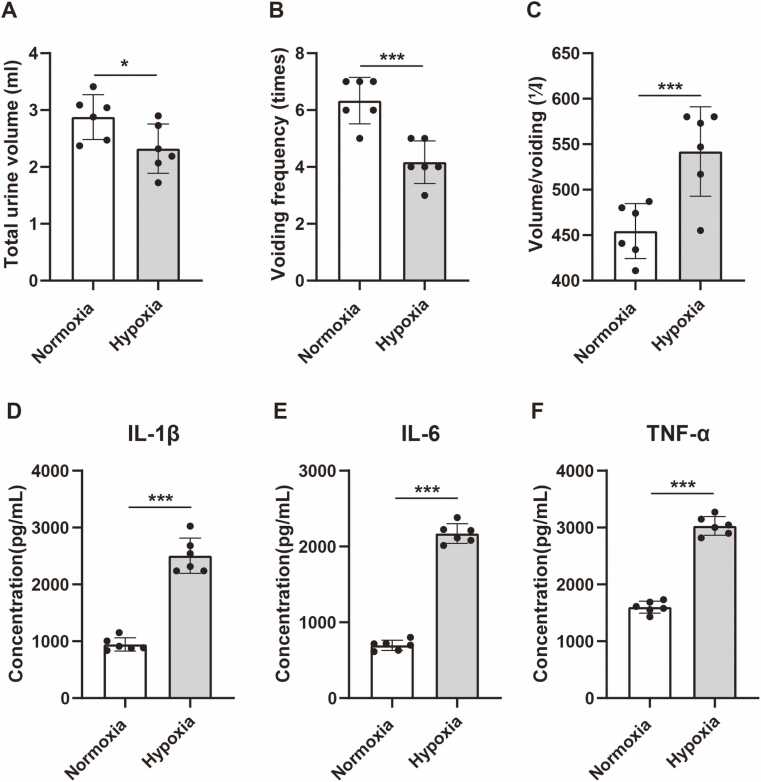
Hypobaric Hypoxia-Induced Urinary Retention and Neuroinflammation. A. Compared to the normoxic group, the mice in HH group showed a significant decrease in total urine volume (2.88 ± 0.39 vs. 2.32 ± 0.44; n = 6 male mice per group; two-sided t test, *p = 0.043). B. The mice in HH group also showed a significant decrease in voiding frequency (6.33 ± 0.82 vs. 4.17 ± 0.75; ***p < 0.001). C. The mice in HH group showed a significant increase in single void volume (454.5 ± 30.27 vs. 542.0 ± 49.13; ***p = 0.004). D. Compared to the normoxic group, the mice in HH group showed a significant upregulation of IL-1β (945.3 ± 116.0 vs. 2506.0 ± 310.1; ***p < 0.001). E. The mice in HH group also showed a significant upregulation of IL-6 (696.8 ± 67.7 vs. 2171.0 ± 129.8; ***p < 0.001). F. The mice in HH group also showed a significant upregulation of TNF-α (1600.0 ± 106.0 vs. 3030.0 ± 165.4; ***p < 0.001). Data are shown as mean±SD.

In this study, we observed the 24-hour urine volume of mice, and took the total urine volume, voiding frequency and single void volume as the basis for evaluating urinary retention. The experimental group mice were placed in HH environment for 48 h, while the control group mice were placed in a normoxic environment for the same time. The results showed that compared with the control group, the total urine volume (Fig. 2 A) and voiding frequency of the experimental group mice (Fig. 2 B) under the condition of HH were significantly reduced, but the single void volume (Fig. 2 C) was significantly increased, showing the symptoms of AUR. After the urine evaluation experiment, we sacrificed the experimental group mice and separately lysed M1 region, and used ELISA method to detect the expression of inflammatory mediators in M1. The results showed that compared with the control group, the experimental group mice showed a significant increase in the expression of IL-1 β (Fig. 2 D), IL-6 (Fig. 2 E), and TNF - α (Fig. 2 F)under HH condition, indicating a link between HH exposure and neuroinflammation.

### Endoplasmic reticulum stress is activated by hypobaric hypoxia and modulated by 4-PBA

**Fig. 3:**
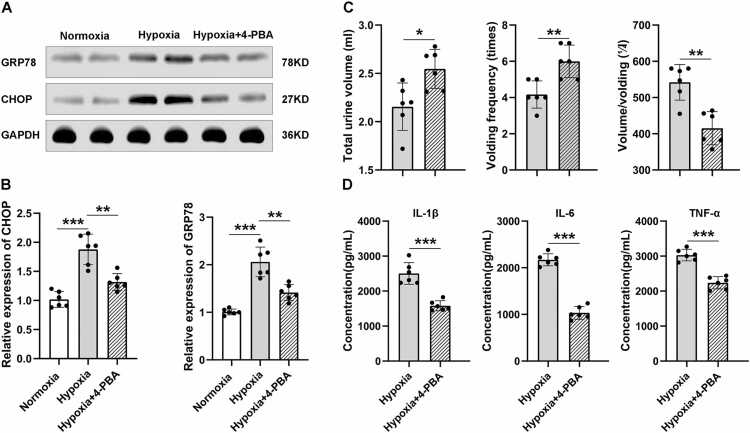
Modulation of Endoplasmic Reticulum Stress and Inflammatory Markers. A. Compared to the normoxic group, M1 region of the mice in HH group showed a significant upregulation of ERS markers CHOP and GRP78, while decreased in hypoxia group treatment with ERS inhibitor 4-PBA. B. West Blot detection showed a significant upregulation of CHOP and GRP78 in M1 region of the mice in HH group (n = 6 male mice per group; two-sided t test; CHOP: 1.02 ± 0.14 vs. 1.88 ± 0.26, ***p < 0.001, GRP78: 1.01 ± 0.06 vs.2.06 ± 0.31, ***p < 0.001), while decreased in hypoxia group treatment with 4-PBA (CHOP: 1.88 ± 0.26 vs. 1.32 ± 0.14, **p = 0.001, GRP78: 2.06 ± 0.31 vs. 1.42 ± 0.17, **p = 0.001). C. Compared to the HH group, the mice in hypoxia group treatment with 4-PBA administration showed relieved symptoms of AUR, including increased total urine volume (2.32 ± 0.44 vs. 2.55 ± 0.20; n = 6 male mice per group; two-sided t test; *p = 0.012) and voiding frequency (4.17 ± 0.75 vs. 6.00 ± 0.89; **p = 0.003), and decreased single void volume (542.0 ± 49.13 vs. 415.3 ± 45.83; **p = 0.001). D. The mice in hypoxia group treatment with 4-PBA administration also showed relieved neuroinflammation, including decreased IL-1β (2506.0 ± 310.1 vs. 1581.0 ± 142.8; ***p < 0.001), IL-6 (2171.0 ± 129.8 vs. 1034.0 ± 137.2; ***p < 0.001)and TNF-α (3030.0 ± 165.4 vs. 2238.0 ± 177.4; ***p < 0.001). Data are shown as mean±SD.

Meanwhile, we also tested the expression of ERS in M1. The results showed that in the experimental group, exposure to HH significantly activated ERS, manifested by a significant increase in ERS markers GRP78 and CHOP, which were not observed in the normoxic group. Furthermore, we administered the ERS inhibitor 4-PBA to the experimental group, which effectively suppressed the ERS induced by HH, as evidenced by the expression of GRP78 (Fig. 3 A) and CHOP (Fig. 3 B) markers. At the same time, administration of 4-PBA also alleviated AUR symptoms, manifested as an increase in total urine volume and voiding frequency, as well as a decrease in single void volume (Fig. 3 C). Finally, we also measured the expression levels of IL-1 β, IL-6, and TNF - α in M1, and the results showed a significant decrease in the expression of these inflammatory mediators, suggesting that administration of 4-PBA can also improve neuroinflammation (Fig. 3 D). These findings suggest that the AUR induced by HH exposure is mediated by ERS, and inhibiting ERS can improve AUR and related neuroinflammation.

### Hypobaric hypoxia causes neuronal damage and apoptosis in M1

**Fig. 4:**
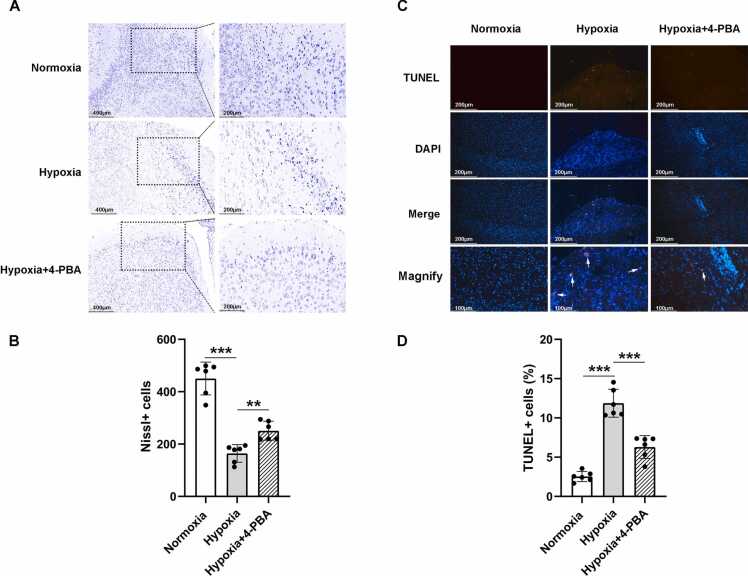
Neuronal Damage and Apoptosis in Response to Hypobaric Hypoxia. A. Nissl staining reveals a significant reduction in the number of neurons M1 region of the mice in HH group compared to the normoxic group, while increased in hypoxia group treatment with ERS inhibitor 4-PBA. B. The number of neurons in M1 of the HH group was significantly less than that in the normoxic group (450.5 ± 62.77 vs. 164.2 ± 33.7; n = 6 male mice per group; two-sided t test; ***p < 0.001), while the number of neurons in hypoxia group treatment with ERS inhibitor 4-PBA was significantly higher than that in the HH group (164.2 ± 33.7 vs. 36.26; **p = 0.0016). C. TUNEL staining indicated increased cell apoptosis compared to normoxia neurons in M1 region of the mice in HH group compared to the normoxic group, while decreased in hypoxia group treatment with ERS inhibitor 4-PBA. D. The number of apoptosis neurons in M1 of the HH group was higher than that in the normoxic group (2.54 ± 0.64 vs. 11.87 ± 1.78; n = 6 male mice per group; two-sided t test; ***p < 0.001), while the number of neurons in hypoxia group treatment with ERS inhibitor 4-PBA was less than that in the HH group (11.87 ± 1.78 vs. 6.29 ± 1.45; ***p < 0.001). Data are shown as mean±SD.

**Fig. 5:**
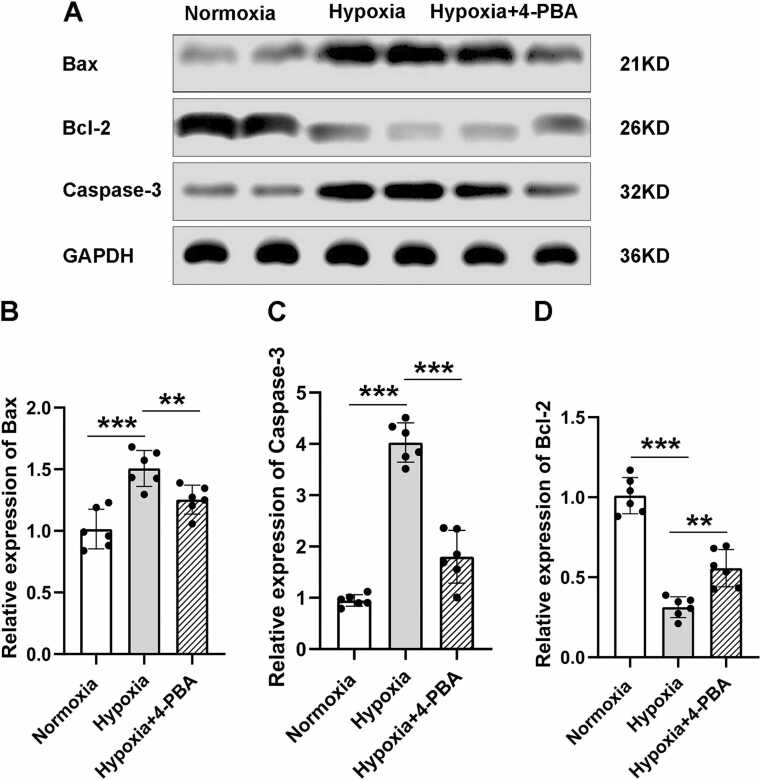
Expression of Apoptosis Markers in Hypobaric Hypoxia. A. Compared to the normoxic group, M1 region of the mice in HH group showed a significant upregulation of pro-apoptotic proteins Bax and caspase-3, and downregulation of anti-apoptotic protein Bcl-2. While, in hypoxia group treatment with ERS inhibitor 4-PBA, this expression alteration has been suppressed. B. West Blot detection showed a significant upregulation of Bax a in M1 region of the mice in HH group (1.02 ± 0.16 vs. 1.51 ± 0.15; n = 6 male mice per group; two-sided t test; ***p < 0.001), while decreased in hypoxia group treatment with 4-PBA (1.51 ± 0.15 vs. 1.25 ± 0.12; **p = 0.0078). C. West Blot detection showed a significant upregulation of caspase-3 in M1 region of the mice in HH group (0.95 ± 0.11 vs. 4.02 ± 0.38; n = 6 male mice per group; two-sided t test; ***p < 0.001), while decreased in hypoxia group treatment with 4-PBA (4.02 ± 0.38 vs. 1.80 ± 0.51; ***p < 0.001). D. West Blot detection showed a significant downregulation of Bcl-2 in M1 region of the mice in HH group (1.01 ± 0.11 vs. 0.31 ± 0.06; n = 6 male mice per group; two-sided t test; ***p < 0.001), while increased in hy*p*oxia group treatment with 4-PBA (0.31 ± 0.06 vs. 0.56 ± 0.12; **p = 0.0012). Data are shown as mean±SD.

Finally, we examined the neuronal damage and apoptosis status after HH exposure. We observed the M1 region using the Nissl staining and found that compared to the normoxic group, the experimental group showed a significant increase in neuronal damage under HH exposure (Fig. 4 A), manifested as a decrease in the number of neurons and karyopyknosis (Fig. 4 B). Furthermore, the results of TUNEL staining also confirmed that under HH exposure (Fig. 4 C), neuronal apoptosis in the M1 region was significantly increased compared to the normoxic state (Fig. 4 D). At the same time, we used Western blot analysis to detect the expression of pro apoptotic and anti apoptotic proteins in the M1 region (Fig. 5 A). Here, we detected the expression of apoptosis related proteins caspase-3, Bax, and Bcl-2. In previous reports, ERS induced apoptosis pathway can lead to an increase in the expression levels of pro apoptotic caspase-3 and Bax proteins, followed by a decrease in the level of anti apoptotic protein Bcl-2 (Zhao et al., 2024, Qureshi et al., 2023). Compared with normoxic exposure, the levels of pro-apoptotic proteins caspase-3 (Fig. 5 B) and Bax (Fig. 5 C) significantly increased under HH exposure, while the expression of anti-apoptotic protein Bcl-2 (Fig. 5 D) decreased, further confirming the neuronal apoptosis effect caused by HH. Similarly, we also administered 4-PBA to inhibit ESR, and the results showed that 4-PBA significantly reduced HH induced neuronal damage and apoptosis, once again confirming that the neurological dysfunction caused by HH exposure is mediated by ERS (Fig. 5 A-D).

## Discussion

This study aimed to investigate the role of ERS in HH-induced AUR. Our findings indicate that HH leads to AUR, endoplasmic reticulum stress, neuroinflammation, and apoptosis. Inhibition of ERS can improve neuroinflammation, reduce apoptosis, and alleviate urinary retention symptoms. This study highlights the mechanisms underlying ERS in HH-induced AUR. As far as we know, this is the only study to explore the role of cerebral cortex in HH-induced AUR at high altitude.

HH, characterized by low atmospheric pressure and reduced oxygen availability, is a significant stressor affecting various physiological functions (Almanza et al., 2019). Previous studies have shown that it impacts urodynamic indicators. Studies from Italy have confirmed that bladder contractility decreases and urination time prolongs in high-altitude environments (Verratti et al., 2016, Verratti et al., 2019). Similarly, a Chinese study reported that first-time entrants to high-altitude areas experienced urinary retention, characterized by decreased urination frequency and volume (Wang, 2012). In these studies, urinary retention was considered as physiological adaptation of the human body exposed to HH, while its internal mechanism remains unknown. However, they also mentioned that the understanding of urinary tract physiology at high altitude should be integrated by the evaluation of other organ systems, such as the nutritional, hormonal, immunological, and neurological systems (Verratti et al., 2019).

**Fig. 6:**
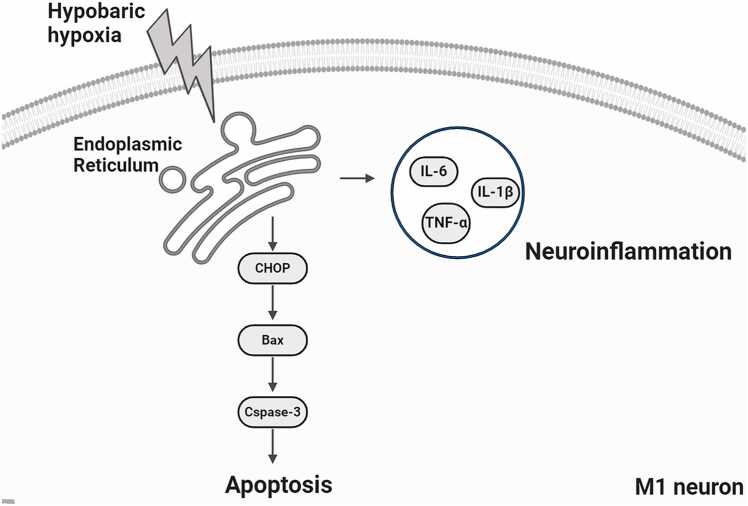
Mechanism diagram of neuroinflammation and neuronal apoptosis induced by ERS due to HH in M1.

A long-standing model for the neural control of urination proposes a two-level system in which a cortical top-down circuit controls a subcortical on–off switching circuit. This circuit allows for a conscious transition from urine storage to voiding (Fowler et al., 2008a, Fowler et al., 2008b, Griffiths and Fowler, 2013, Manohar et al., 2017). Extensive studies previously have centered on a subcortical reflex circuitry operating from structures including the lower urinary tract, the spinal cord, the pontine micturition center, and the periaqueductal gray (de Groat and Wickens, 2013, Griffiths, 2004). But the exact location and identity of urination-related cortical neurons have never before been causally demonstrated. Until we confirmed in our study that neurons in M1 project directly to the bladder, and their activation triggers bladder contraction and urination (Yao et al., 2018). Inhibition or damage to these neurons will lead to urinary retention. This result confirms the direct projection between cortical neurons and the bladder, as well as their capacity to control urination (Fig. 6).

Previous research has shown that hypoxia can induce ERS and apoptosis in various cell types, including neuronal cells. For instance, Karar et al. (2008) reported that acute HH led to the downregulation of ER chaperones in murine kidneys, suggesting a reduced capacity to cope with protein folding stress. Yuan et al. (2015) demonstrated that chronic intermittent HH ameliorates ERS-mediated liver damage in fructose-fed rats, indicating that HH can exacerbate ERS in metabolic tissues. In this study, we investigated whether ERS plays a role in HH-induced AUR, focusing on the urinary control brain region M1. The results showed that ERS markers GRP78 and CHOP were significantly upregulated in the hypoxic group, indicating that low-pressure hypoxia triggers ERS in M1 neurons. At the same time, neuronal damage, apoptosis and the expression of apoptotic proteins also increased. Conversely, inhibiting ER stress with 4-PBA reduced nerve injury, neuroinflammation, and apoptosis, and relieved urinary retention symptoms. These results reveal that acute urinary retention and the endoplasmic reticulum stress of neurons are closely related.

However, as with any research, there are limitations that must be acknowledged and areas for future exploration. Firstly, while our study establishes a correlation between HH and AUR, it is limited by the use of a single animal model. The translation of these findings to diverse populations and other species requires further investigation. Secondly, the study's focus on the M1, although novel, does not capture the full spectrum of neural centers involved in micturition. Future studies should consider examining other regulatory centers such as the pontine micturition center and the periventricular gray matter to gain a comprehensive understanding. Finally, this study did not explore the changes in urodynamics after HH, which are crucial for understanding the progress of AUR.

In conclusion, this study provides a foundation for understanding the role of ERS in HH-induced AUR and opens up potential avenues for therapeutic intervention. However, more research is needed to validate these findings in human studies and to investigate other ERS pathways that might be involved. Additionally, combining ERS inhibition with other neuroprotective strategies could enhance treatment efficacy for AUR in high-altitude settings.

## Author contributions

QCZ and YYM carried out animal modeling, Nissl staining, TUNEL staining, ELISA assay and WB assays. CBL, LN and HWC carried out animal modeling, Nissl staining and TUNEL staining. QCZ, YYM, JJC, JHZ, and YHH participated in the design of the study. QCZ and YYM performed the statistical analysis. QCZ and YHH drafted the manuscript. All authors contributed to the article and approved the submitted version.

## CRediT authorship contribution statement

**Yinghui Huang:** Writing – review & editing, Funding acquisition, Conceptualization. **Yingying Ma:** Writing – original draft, Data curation. **Quanchao Zhang:** Writing – original draft, Funding acquisition, Formal analysis, Data curation, Conceptualization. **Ling Nie:** Data curation. **Caibao Lu:** Data curation. **Jiujian Cao:** Data curation. **Hongwei Chen:** Data curation. **Jinghong Zhao:** Writing – review & editing, Conceptualization.

## Ethics approval and consent to participate

All experiments protocol involving animals were reviewed and approved by the Laboratory Animal Welfare and Ethics Committee of Third Military Medical University, and were in strict accordance with the National Institutes of Health Guidelines for the Care and Use of Laboratory Animals.

## Funding

This work was supported by grants from the 10.13039/501100001809National Natural Science Foundation of China [NO. 82101967] and Army Medical University Excellent Talent Pool (Key Support Objects).

## Conflicts of Interest

No potential conflict of interest was reported by the authors.

## Data Availability

The data and materials supporting the results in this article are available from the corresponding author on reasonable request.
